# Shared molecular signatures between coronavirus infection and neurodegenerative diseases provide targets for broad-spectrum drug development

**DOI:** 10.1038/s41598-023-29778-4

**Published:** 2023-04-04

**Authors:** Li Deng, Ling Ding, Xianlai Duan, Yousong Peng

**Affiliations:** 1 Internal Medicine-Neurology, The Third Hospital of Changsha, Changsha, 410015 China; 2 Bioinformatics Center, College of Biology, Hunan Provincial Key Laboratory of Medical Virology, Hunan University, Changsha, 410082 China

**Keywords:** Data integration, Data mining, Gene ontology, SARS-CoV-2, SARS virus, Neurodegenerative diseases

## Abstract

Growing evidences have suggested the association between coronavirus infection and neurodegenerative diseases. However, the molecular mechanism behind the association is complex and remains to be clarified. This study integrated human genes involved in infections of three coronaviruses including SARS-CoV-2, SARS-CoV and MERS-CoV from multi-omics data, and investigated the shared genes and molecular functions between coronavirus infection and two neurodegenerative diseases, namely Alzheimer’s Disease (AD) and Parkinson’s Disease (PD). Seven genes including HSP90AA1, ALDH2, CAV1, COMT, MTOR, IGF2R and HSPA1A, and several inflammation and stress response-related molecular functions such as MAPK signaling pathway, NF-kappa B signaling pathway, responses to oxidative or chemical stress were common to both coronavirus infection and neurodegenerative diseases. These genes were further found to interact with more than 20 other viruses. Finally, drugs targeting these genes were identified. The study would not only help clarify the molecular mechanism behind the association between coronavirus infection and neurodegenerative diseases, but also provide novel targets for the development of broad-spectrum drugs against both coronaviruses and neurodegenerative diseases.

Neurodegenerative diseases are caused by the progressive loss of structure or function of neurons which is often age-dependent. They become more and more prevalent with the rapid increase of the elder in the world, and pose a major threat to human health^1,2^. Neurodegenerative diseases include several diseases such as amyotrophic lateral sclerosis, Alzheimer’s disease (AD), Parkinson’s disease (PD), Huntington’s disease, prion diseases, and so on. Among them, AD and PD are most commonly observed. For example, there is an estimated 6.2 million Americans age 65 and older living with Alzheimer's dementia as of 2021^3^. AD and PD often cause severe or even fatal dementia as the diseases progress, which significantly decreases the quality of life for patients and their caregivers and brings a heavy economic burden to society^4^. Unfortunately, there is still a lack of effective drugs against these diseases although several drugs have been approved for their treatment^5^.

Many factors such as genetic mutation, environmental exposure to toxins or viruses or other contaminating agents have been reported to influence the occurrence of neurodegenerative diseases^2,6,7^. Among them, researchers have long suspected the involvement of viral infections in the onset or progression of neurodegenerative diseases, especially the AD and PD^7–11^. One of the most famous yet controversial examples is the outbreaks of parkinsonism that occurred following the 1918 influenza pandemic^7^. Several viruses have been suggested to be associated with AD or PD. The neurotropic viruses such as the arboviruses, influenza viruses and herpes viruses can directly infect the central nervous system (CNS) or elicit CNS inflammation which may cause neuronal damage or death^7,9^. While other viruses such as the Coxsackie virus and HIV can induce the cytokine storm in the brain which can be long-lasting and cause insult later in the life^7^.

The coronavirus is the largest kind of RNA virus with genome ranging from 26 to 32 kb. More than 40 coronavirus species have been identified. Until to date, seven coronaviruses have been reported to infect humans, among which the SARS-CoV-2, SARS-CoV and MERS-CoV are most-deadly and have caused enormous morbidity and mortality to humans^12^. For example, the COVID-19 that is caused by SARS-CoV-2 has resulted in 486,761,597 human infections and 6,142,735 human deaths worldwide as of April 1st, 2022^13^. The three deadly coronaviruses mentioned above have been reported to infect multiple organs or tissues in humans or mice, and be associated with various disease comorbidities and complications^14–18^. Especially, a large proportion of COVID-19 patients have developed neurological symptoms which were termed as NeuroCOVID^19^. Some patients even developed the parkinsonism after the SARS-CoV-2 infection^11^. Besides SARS-CoV-2, patients infected by MERS-CoV and SARS-CoV were also reported to present severe neurological symptoms or complications^20,21^. This suggests potential associations between coronavirus infection and neurodegenerative diseases.

Actually, lots of studies further provided evidences supporting the associations between coronavirus infection and neurodegenerative diseases. Several studies in mice or non-human primate models have shown that multiple coronaviruses such as SARS-CoV-2 and SARS-CoV can invade the CNS through the olfactory bulb and further spread to functional areas of the CNS such as the hippocampus and thalamus^11,22^. Studies on humans further showed the existence of viral RNA, protein or particles in the brain of SARS-CoV or SARS-CoV-2 patients. For example, Puelles et al. observed a small amount of virus RNA and proteins in 8 of 22 COVID-19 patients in autopsy studies^17^. Paniz-Mandolfifi et al. reported the presence of SARS-CoV-2 in neural and capillary endothelial cells in frontal lobe tissue obtained at postmortem examination from a COVID-19 patient^23^. Besides, the coronavirus infection can also elicit the cytokine storm which may cause damage to CNS indirectly^11^.

The molecular mechanisms behind the association between coronavirus infection and neurodegenerative diseases are complex. The virus proteins can interact with human proteins which may be involved in diseases. For example, the human proteins which interact with SARS-CoV-2 proteins were also involved in several biological processes related to aging and neurodegenerative diseases^18,24^, such as dysfunction of protein homeostasis and mitochondrial function, responses to oxidative stress, lipid metabolism. However, there is still a lack of systematic study about the human genes and molecular functions shared between coronavirus infection and neurodegenerative diseases. This study for the first time integrated human genes involved in infections of three coronaviruses, i.e., SARS-CoV-2, SARS-CoV and MERS-CoV, from multi-omics data, and investigated the shared genes and molecular functions between coronavirus infection and two neurodegenerative diseases, i.e., AD and PD. Seven genes and dozens of molecular functions were found to play important roles in both coronavirus infection and neurodegenerative diseases. These genes were also found to interact with more than 20 other viruses. Finally, drugs targeting these genes were identified which may have effects on both virus infection and neurodegenerative diseases.

## Result

### Overview of the study

**Figure 1:**
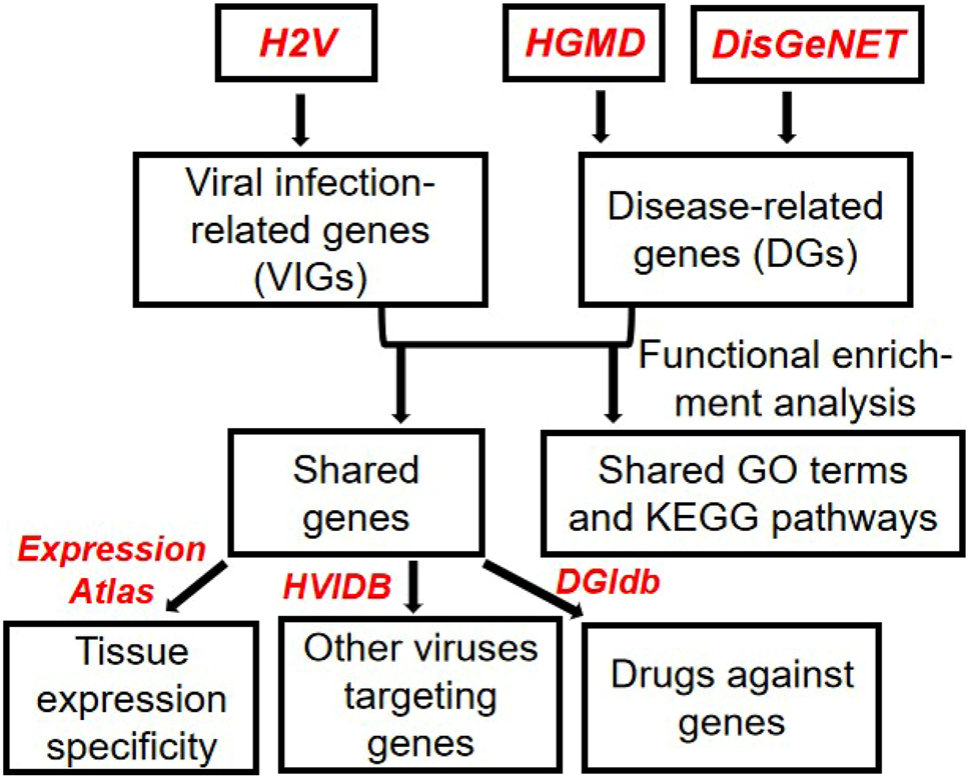
The workflow of the study. The database names were shown in italic and colored in red.

The workflow of the study was shown in Fig. 1. Firstly, the viral infection-related genes (VIGs) for three coronaviruses including SARS-CoV-2, SARS-CoV and MERS-CoV, and disease-related genes (DGs) for AD and PD were obtained from databases of H2V, HGMD and DisGeNET, respectively. Then, the common genes and molecular functions between coronavirus infection and neurodegenerative diseases were obtained. Finally, the tissue expression specificity of the common genes were analyzed based on the Expression Atlas database; the interactions between these genes and other viruses besides coronaviruses were analyzed based on protein–protein interactions derived from the HVIDB database; the drugs targeting these genes were obtained from the DGIdb database.

### Integration of viral infection-related and disease-related genes from multi-omics data

The VIGs were obtained for three deadly coronaviruses including the SARS-CoV-2, SARS-CoV and MERS-CoV from the H2V database (Table S1) (Materials and Methods). The VIGs included seven kinds of genes from multi-omics data: the differentially expressed genes (DEGs), differentially expressed proteins (DEPs), differentially phosphorylated proteins (DPPs), differentially translated proteins (DTPs), differentially ubiquitinated proteins (DUPs), proteins that participate in human-virus protein–protein interactions (P-PPIs), and disease severity associated proteins (SAPs) (Table 1). For the SARS-CoV-2, VIGs included 810 DEGs, 171 DEPs, 1,987 DPPs, 111 DTPs, 267 DUPs, 1,257 P-PPIs and 335 SAPs. Only a few genes overlapped between different kinds of VIGs (Table S2). For example, only 13 genes were observed in both the DEGs and DEPs. This suggested that different kinds of VIGs capture complementary aspects of the viral infection. Therefore, seven kinds of VIGs from multi-omics data were incorporated for a better understanding of the pathogenesis of coronavirus infection.

**Table 1:** The number of different kinds of VIGs in SARS-CoV-2, SARS-CoV and MERS-CoV.

Virus	DEG	DEP	DPP	DTP	DUP	P-PPI	SAP	Total
SARS-CoV-2	810	171	1987	111	267	1257	335	4228
SARS-CoV	1487	51	–	–	–	998	–	2488
MERS-CoV	8895	–	–	–	–	292	–	9038

*DEG* differentially expressed genes; *DEP* differentially expressed proteins; *DPP* differentially phosphorylated proteins; *DTP* differentially translated proteins; *DUP* differentially ubiquitinated proteins; *P-PPI* proteins that participate in human-virus protein–protein interactions; *SAP* disease severity associated proteins.

For the SARS-CoV, 2,488 VIGs were obtained which included 1,487 DEGs, 51 DEPs and 998 P-PPIs; for the MERS-CoV, 9,038 VIGs were obtained which included 8,895 DEGs and 292 P-PPIs (Table 1). Only a few genes overlapped between different kinds of VIGs for both SARS-CoV and MERS-CoV.

The DGs for both AD and PD were obtained from the HGMD and DisGeNET databases (Materials and Methods). A total of 886 DGs for AD were obtained which included 105 and 852 genes obtained from the HGMD and DisGeNET databases, respectively (Table S1). A total of 481 DGs for PD were obtained which included 85 and 450 genes obtained from the HGMD and DisGeNET databases, respectively (Table S1).

### The inflammation and stress response-related molecular functions were common in coronavirus infection and AD

**Figure 2:**
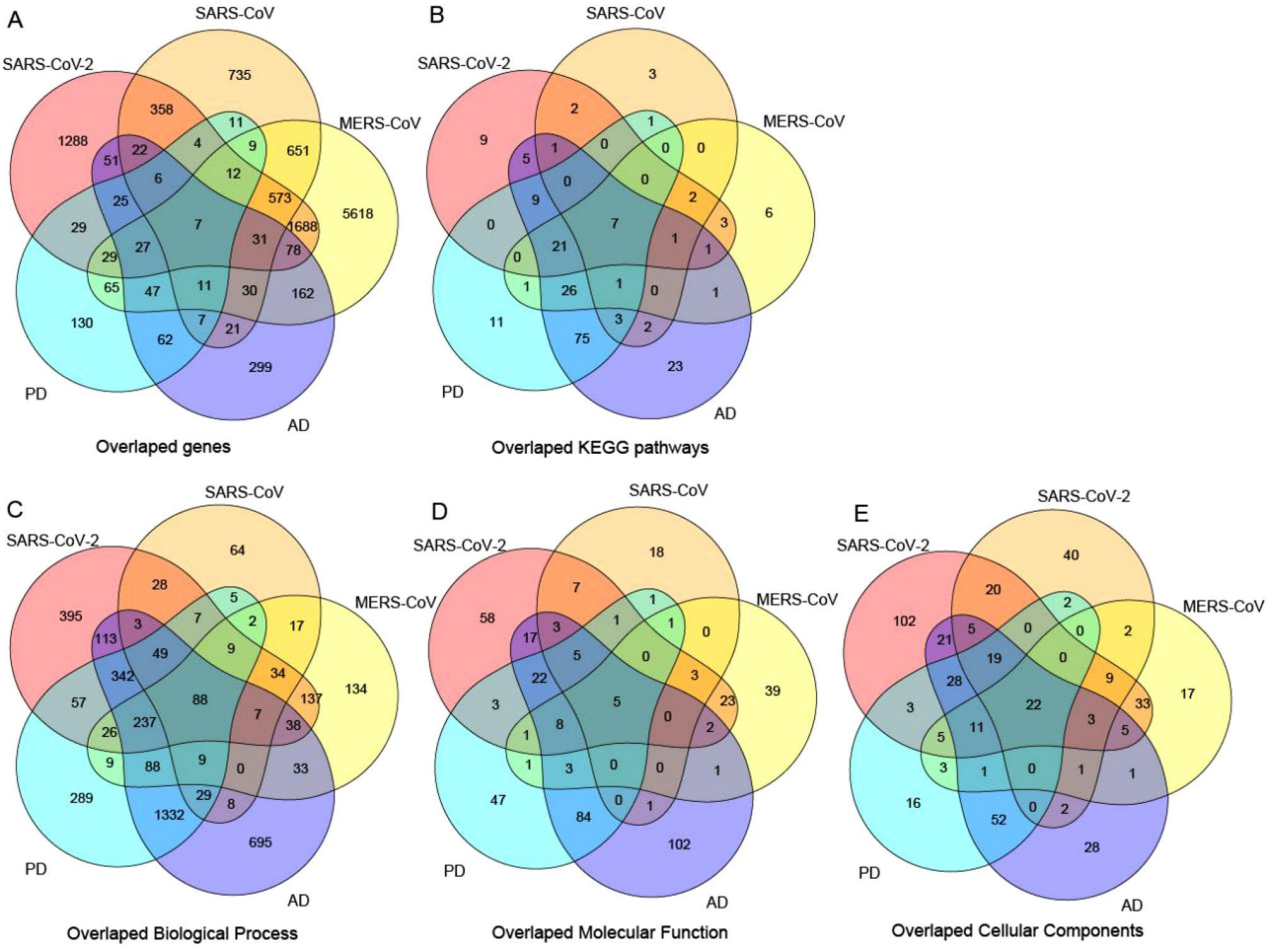
The common molecular signatures between three coronaviruses (SARS-CoV-2, SARS-CoV and MERS-CoV) and two neurodegenerative diseases (AD and PD). (**A**) The overlapped genes between VIGs and DGs; (**B**–**E**) The overlapped KEGG pathways (**B**) and GO terms in domains of Biological Process (**C**), Molecular Function (**D**) and Cellular Component (**E**).

The enriched gene ontology (GO) terms and KEGG pathways in the VIGs of three coronaviruses and DGs of two neurodegenerative diseases were obtained firstly (Table S3). Then, the common genes and functions between VIGs of all three coronaviruses and DGs of AD were analyzed. 38 genes were found to be involved in infection of three coronaviruses and AD (Fig. 2 and Table S4). A total of 8 KEGG pathways, and 95, 5, 25 GO terms in the domain of Biological Process, Molecular Function, Cellular Component, respectively, were observed between the enriched functions in VIGs of three coronaviruses and DGs of AD (Fig. 2 and Table S5).

**Figure 3:**
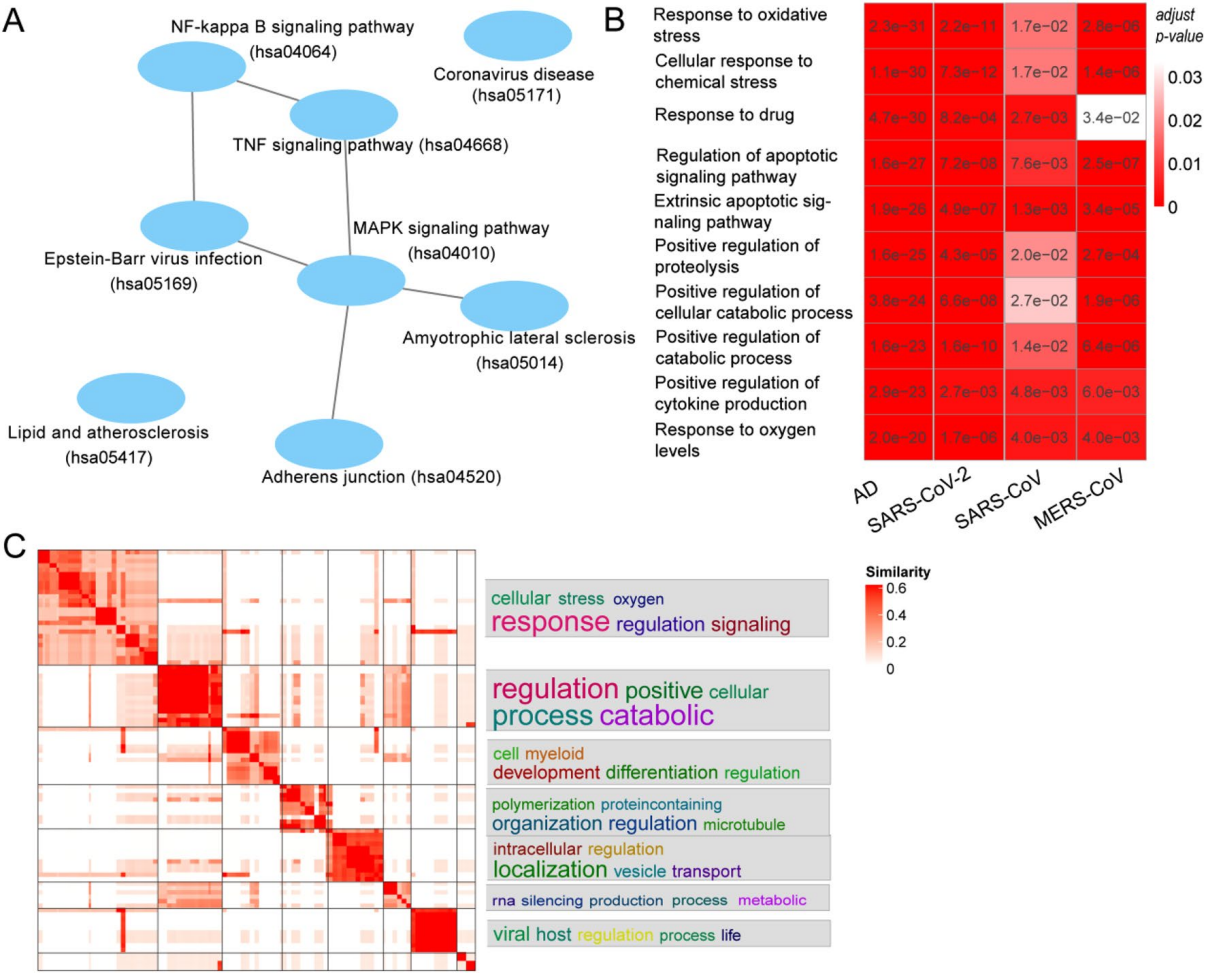
The shared KEGG pathways and biological processes between coronavirus infection and AD. (**A**) The shared KEGG pathways between coronavirus infection and AD^25^. (**B**) Top ten most enriched GO terms in the domain of Biological Processes. The GO terms were sorted by the adjusted p-value of GO terms obtained in AD. The heat map was colored based on the adjusted p-value (shown in the heat map) according to the legend in the top right. (**C**) Clustering of the shared GO terms in the domain of Biological Process between coronavirus infection and AD. The heat map was colored based on the pairwise similarity of GO terms according to the figure legend in the top right. The words in the right side of the figure referred to top five words which appeared most frequently in the GO terms of the corresponding cluster that were shown in the heat map. The size of words was in proportion to the frequency of them in GO terms of the corresponding cluster.

In the shared KEGG pathways, six pathways were connected in the pathway network (Fig. 3A). Three pathways including the “MAPK signaling pathway”, “TNF signaling pathway” and “NF-kappa B signaling pathway” were reported to be involved in inflammation and immune response. Among them, the “MAPK signaling pathway” was the hub node in the pathway network and connected to four pathways. Interestingly, two virus infection-related pathways including “Coronavirus disease” and “Epstein-Barr virus infection” were enriched in DGs of AD, while two neurodegenerative disease-related pathways including “Amyotrophic lateral sclerosis” and “Lipid and atherosclerosis” were enriched in VIGs of three coronaviruses.

In the shared GO terms in the domain of Biological Process, when analyzing the top ten GO terms, three of them were related to response to oxidative or chemical stress and five of them were related to regulation of biological processes such as signaling and catabolic process (Fig. 3B). When analyzing all 95 GO terms, they were grouped into 9 clusters (Fig. 3C). The largest cluster was related to responses to signaling, stress, regulations, and so on; the second cluster was related to regulation of biological processes such as signaling and catabolic process; other clusters were related to development, organization, localization, RNA silencing, viral infection, and so on (Fig. 3C). In the shared GO terms in the domain of Molecular Function, all five GO terms were binding-related such as “ubiquitin protein ligase binding” and “chaperone binding” (Table S6). In the shared GO terms in the domain of Cellular Component, most GO terms were related to vesicle-like structures in the cytoplasm such as the vesicle, granule, endosome, vacuole, and so on (Table S6).

### Common molecular signatures between coronavirus infection and PD were also observed in AD

Then, the common genes and functions between VIGs of all three coronaviruses and DGs of PD were analyzed. 19 genes were found to be involved in the infection of three coronaviruses and PD (Fig. 2 and Table S4). A total of 7 KEGG pathways, and 97, 5, 22 GO terms in the domain of Biological Process, Molecular Function, Cellular Component, respectively, were observed between the enriched functions in VIGs of three coronaviruses and DGs of PD (Fig. 2 and Table S5). Interestingly, most of the enriched functions shared between coronaviruses and PD were also found in AD (Fig. 2 and Table S5). For example, 100%, 88%, 89% and 91% of enriched KEGG pathways, GO terms in the domain of Biological Process, Molecular Function, Cellular Components shared between the SARS-CoV-2 and PD were also found in AD (Table S5).

### Seven genes were involved in infections of three coronaviruses and two neurodegenerative diseases

**Figure 4:**
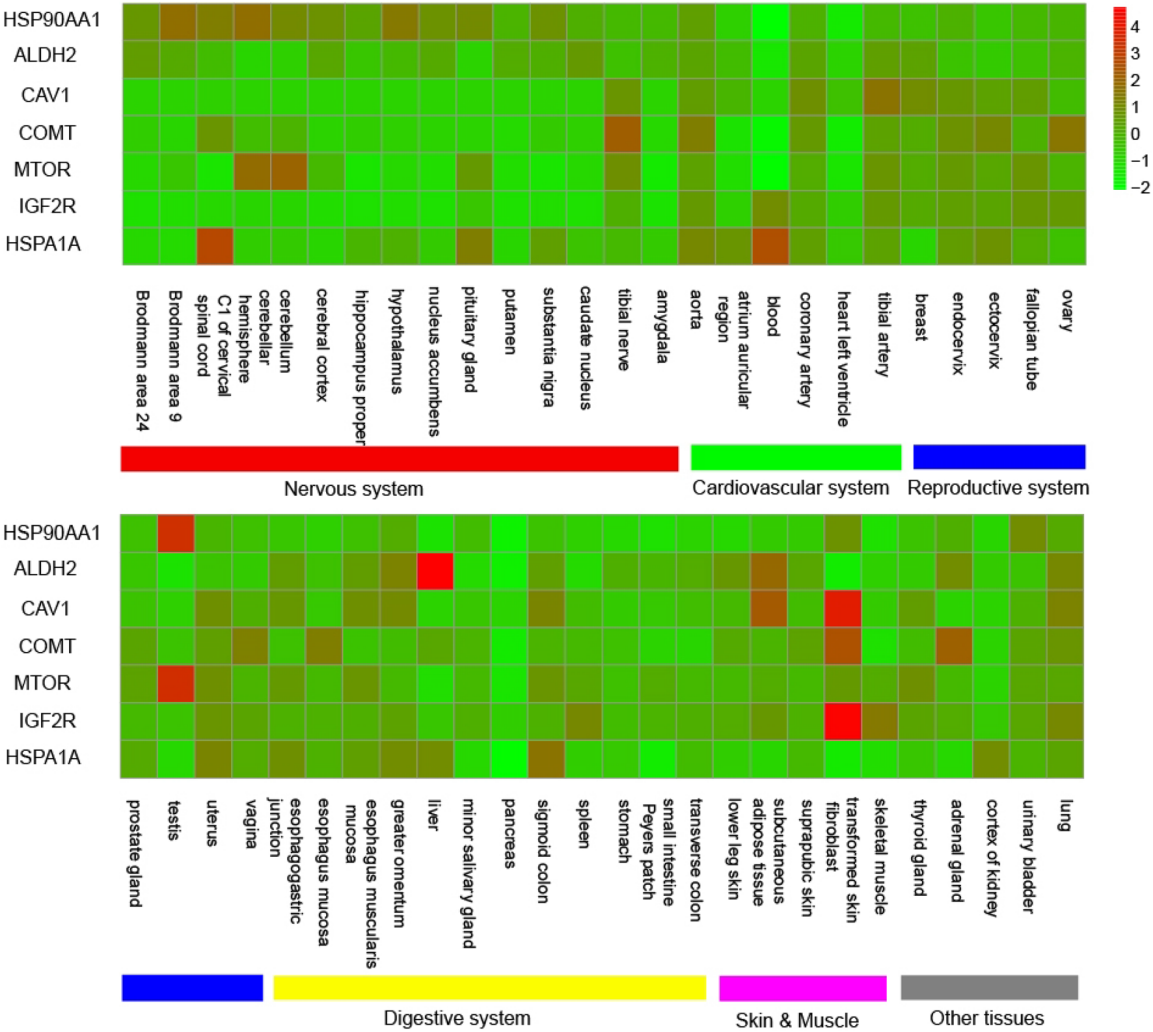
The expression of seven genes in 52 human tissues. The heat map listed the normalized expression value of genes in tissues according to the legend in the top right. The genes were shown in the left side of the figure. The tissues were shown at the bottom of the figure and were organized by systems (colored bars).

The common molecular signatures between three coronaviruses and two neurodegenerative diseases were further analyzed. Seven genes were observed to be involved in both infections of three coronaviruses and two neurodegenerative diseases, including the HSP90AA1, ALDH2, CAV1, COMT, MTOR, IGF2R and HSPA1A (Table 2). The expression specificity of these genes in human tissues were analyzed. As shown in Fig. 4, the HSP90AA1 had medium to high expression in several brain tissues such as the Brodmann area 9, cerebellar hemisphere and hypothalamus; the COMT, MTOR and HSPA1A had high expression in one or two brain tissues such as cerebellum; other three genes had low to medium expression in brain tissues. In the lung where three coronaviruses infect, all seven genes had medium expression.

**Table 2:** Seven genes which were involved in both infections of three coronaviruses and two neurodegenerative diseases, and the drug targeted against these genes.

Gene	Gene full name	Num of drugs	Top 1 drug*	Mol. type of drug	Score^#^
HSP90AA1	Heat shock protein 90 alpha family class A member 1	3	ARGENTEOSIDE A	–	0.94
ALDH2	Aldehyde dehydrogenase 2 family member	5	PRUNETIN	–	12.73
CAV1	Caveolin 1	2	TESTOSTERONE	Small Molecule	1.77
COMT	Catechol-O-methyltransferase	12	ENTACAPONE	Small Molecule	16.91
MTOR	Mechanistic target of rapamycin kinase	4	RIDAFOROLIMUS	Small Molecule	1.13
IGF2R	Insulin like growth factor 2 receptor	1	MANNOSE 6-PHOSPHATE	–	127.3
HSPA1A	Heat shock protein family A (Hsp70) member 1A	1	CARBAMAZEPINE	Small Molecule	2.71

“-”, no data available.

“*”, the drug with the top interaction score.

“#”, the gene-drug interaction scores provided in the DGIdb database which reflect the confidence and specificity of the gene-drug interactions. The larger, the better. Please see Table S7 for more gene-drug interactions.

A total of 7 KEGG pathways, and 88, 5, 22 GO terms in the domain of Biological Process, Molecular Function, Cellular Component, respectively, were observed between the enriched functions in VIGs of three coronaviruses and DGs of two neurodegenerative diseases (Fig. 2 and Table S5).

### Seven genes were involved in infection of multiple viruses

**Figure 5:**
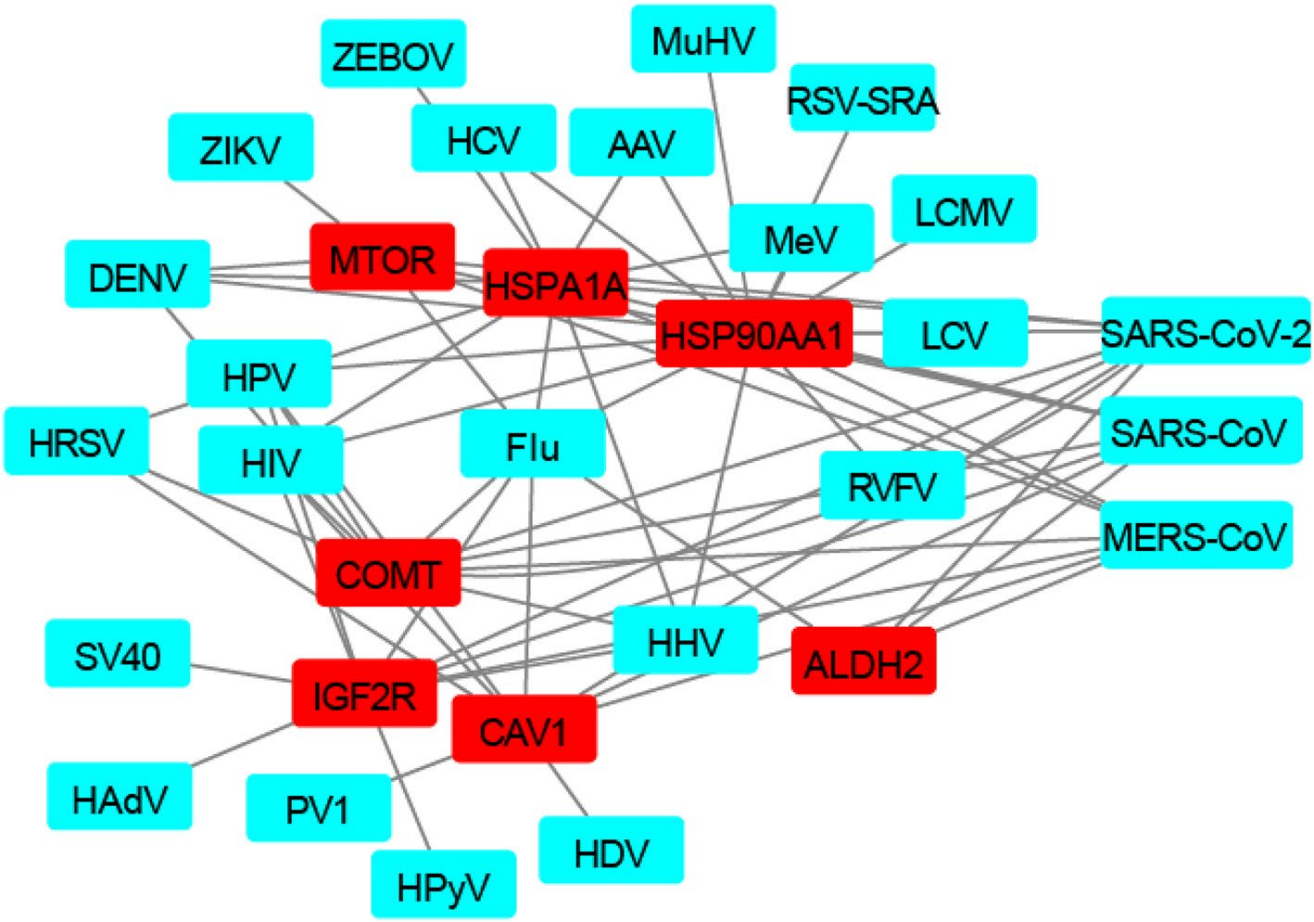
The interactions between seven genes (red) and viruses (light green). The interactions between three coronaviruses (SARS-CoV-2, SARS-CoV and MERS-CoV) and genes were obtained based on this study, while other interactions were obtained based on PPIs between virus and human (Table S8). The full name of these viruses was listed in Table S9.

The seven genes identified here can be taken as targets for the development of drugs that may have a broad-spectrum effect on both coronavirus infection and neurodegenerative diseases. Their roles in infections of other viruses were further investigated. Except three coronaviruses used in the study, a total of 21 viruses were reported to interact with these genes based on protein–protein interactions (PPIs) between viral and human proteins (Fig. 5 and Table S8). Notably, the HSP90AA1 and HSPA1A took part in infections of 16 and 12 viruses, respectively. Then, these genes were mapped to public compounds by searching the DGIdb database for drugs targeting these genes. Overall, a total of 28 high-confidence gene-drug interactions were obtained (Table S7). Most genes were targeted by no more than 5 drugs. Interestingly, the COMT could be targeted by more than 10 drugs (Table 2).

## Discussion

This study for the first time systematically investigated the common genes and molecular functions between coronavirus infections and neurodegenerative diseases. Notably, seven genes were involved in infections of three deadly coronaviruses and two neurodegenerative diseases. They may play an important role in infections of coronaviruses and neurodegenerative diseases. For example, the HSP90 protein that is a molecular chaperone and maintains protein homeostasis during cellular stress has been reported to facilitate infection of SARS-CoV-2^26^, and help protein aggregation and toxic aggregate accumulation in neurodegenerative diseases^27^; the ALDH2 that is crucial in the oxidative metabolism of toxic aldehydes in the brain has been reported to be associated with neurodegenerative diseases^28^ and attenuated immune system to SARS-CoV-2 vaccination^29^. Besides coronaviruses, these genes also play important roles in other viruses (Fig. 5). For example, the HSP90AA1 took part in infections of 13 viruses. It was also reported to be involved in replications of multiple viruses such as Chikungunya Virus, human cytomegalovirus, Human immunodeficiency virus, Hepatitis C virus, Hepatitis B virus, Hepatitis E virus, Herpes Simplex Virus 1, Vaccinia virus, Influenza virus, Rotavirus, and so on^30^, and can induces autophagy in viruses such as the influenza virus^31^. This suggests that drugs identified to target these genes may have broad-spectrum effects on both virus infections and neurodegenerative diseases.

Multiple viruses were reported to be associated with neurodegenerative diseases^7,9,32,33^. The molecular mechanisms behind these associations remain to be learned. A previous study investigated the mechanism of associations between Herpesviridae infection and neurodegenerative diseases, and found that the Oxidative Stress Defense System and LRRK2 pathways (related to inflammation regulation in response to different pathological stimuli) were shared between Herpesviridae infection (cytomegalovirus, Epstein-Bar virus and human herpesvirus 6) and two neurodegenerative diseases (AD and PD)^9^. This study focused on the association mechanism between coronavirus infection and neurodegenerative diseases, and found that several inflammation and stress response-related molecular functions such as the MAPK signaling pathway, NF-kappa B signaling pathway, responses to oxidative or chemical stress were common to both coronavirus infections and neurodegenerative diseases (Fig. 3). Interestingly, both the functions of response to oxidative stress and inflammation were involved in infections of coronavirus and Herpesviridae, and the neurodegenerative diseases, suggesting that these molecular functions may play important roles in the associations between viral infection and neurodegenerative diseases.

Currently, there is a lack of effective drugs for coronavirus infection and neurodegenerative diseases. For the antiviral drugs, most were developed to target the viral proteins that mutate rapidly, which leads to drug resistance frequently^34–36^. On the contrary, the drugs targeting the host protein may have the advantage of stable effect since the host proteins generally evolve far slower than viral proteins^37,38^. Besides, some host proteins may interact with multiple viruses, such as HSP90AA1 and HSPA1A mentioned above. The drugs targeting them may have broad-spectrum antiviral effects. Moreover, by identifying the host genes involved in both viral infection and diseases, the drugs targeting them may have broad-spectrum effects on both viral infection and diseases^9^. Therefore, this study provides a new strategy for the development of drugs against human diseases which have associations with viral infections such as AD and PD.

## Limitation of the study

There were several limitations to the study. Firstly, only two or three kinds of VIGs were obtained for SARS-CoV and MERS-CoV, compared to seven kinds of VIGs for SARS-CoV-2. Besides, the source of VIGs was biased as most VIGs were from the DEG or P-PPI. More data should be incorporated to improve the study in the future. Secondly, the drugs shown in the study were directly obtained from the DGIdb database. The effectiveness of them on coronavirus infection and neurodegenerative diseases needs further studies.

## Conclusion

This study for the first time identified common genes and molecular functions shared between coronavirus infection and neurodegenerative diseases, and further identified drugs targeting the common genes. It helps clarify the molecular mechanism behind the association between coronavirus infection and neurodegenerative diseases, and provides novel targets for the development of broad-spectrum drugs against both coronavirus infection and neurodegenerative diseases.

## Materials and methods

### Data used in the study

The genes involved in the infection of three coronaviruses, i.e., SARS-CoV-2, SARS-CoV and MERS-CoV, were obtained from the H2V database on March 12th, 2022^39^. For genes obtained based on the differential analysis, only those with log2 fold change (Log2FC) greater than 1 and p-value smaller than 0.01 (if available) were kept in analysis (Table S1).

The genes associated with two neurodegenerative diseases, i.e., AD and PD, were obtained from two sources (Table S1): the first is Zhou’s study^18^ which compiled genes that had at least one reported mutation associated the diseases from the Human Gene Mutation Database (HGMD); the other is the DisGeNet database^40^. The keyword of “Alzheimer's disease” (Disease ID: C0002395) or “Parkinson’s disease” (Disease ID: C0030567) was searched against the DisGeNet database on March 15th, 2022. The gene-disease interactions with association score greater than 0.05 were obtained.

The experimentally-determined protein–protein interactions between viruses and humans were obtained from the HVIDB database on February 22th, 2022^41^. The viruses which interacted with each human protein were compiled.

### Functional enrichment analysis of genes

The KEGG pathway and GO enrichment analysis was conducted with functions of *enrichKEGG* and *enrichGO* in the package clusterProfiler (version 3.18.1) in R (version 4.0.3)^42^. All the KEGG pathways and GO terms with FDR adjusted p-values less than 0.05 were considered as significant enrichment. The GO terms were clustered with the simplifyEnrichment package in R^43^.

### Tissue-specific expression of human genes

The expression of human genes in 53 human tissues were obtained from the RNA-Seq data of the Genotype-Tissue Expression (GTEx) Project which were provided in the Expression Atlas database^44^. The tissue of “EBV-transformed lymphocyte” was removed from the analysis as it was not a natural human tissue. The expression level was measured in TPM. To clearly show the tissue-specificity of gene expressions, the expression of each gene in 52 tissues was normalized with the Z-score method.

### Drug repurposing analysis

The drug is defined as any substance that are used as a medication or in the preparation of medication. The drugs which targeted human genes were obtained by searching the DGIdb database with the human gene name on March 30th, 2022^45^. To ensure high confidence and strong interaction specificity between drugs and genes, only the gene-drug interactions with the interaction score greater than 0.5 were kept.

### Visualization of interactions between viruses and human genes

Cytoscape (version 3.8.0) was used to visualize the interactions between viruses and human genes^46^.

## Supplementary Information

Supplementary Information 1.

Supplementary Information 2.

Supplementary Information 3.

Supplementary Information 4.

Supplementary Information 5.

## Data Availability

All data used in the study were available in supplementary materials.
