# Supplementary material for: Shared molecular signatures between coronavirus infection and neurodegenerative diseases provide targets for broad-spectrum drug development
Source: Sci Rep. 2023 Apr 4;13:5457. doi: 10.1038/s41598-023-29778-4 (PMC10071237; doi:10.1038/s41598-023-29778-4)
Supplement: Supplementary file 2 — Supplementary Information 2. [file 41598_2023_29778_MOESM2_ESM.docx]

**Table S2.** Overlap between different kinds of VIGs for the SARS-CoV-2. DEG, differentially expressed genes; DEP, differentially expressed proteins; DPP, differentially phosphorylated proteins; DTP, differentially translated proteins; DUP, differentially ubiquitinated proteins; P-PPI, proteins that participate in human-virus protein–protein interactions; SAP, disease severity associated proteins.

|  | **DEG** | **DEP** | **DPP** | **DTP** | **DUP** | **P-PPI** | **SAP** |
| --- | --- | --- | --- | --- | --- | --- | --- |
| **DEG** | - | 13 | 89 | 5 | 11 | 55 | 4 |
| **DEP** | 13 | - | 28 | 8 | 5 | 30 | 1 |
| **DPP** | 89 | 28 | - | 37 | 85 | 211 | 3 |
| **DTP** | 5 | 8 | 37 | - | 13 | 20 | 1 |
| **DUP** | 11 | 5 | 85 | 13 | - | 50 | 3 |
| **P-PPI** | 55 | 30 | 211 | 20 | 50 | - | 0 |
| **SAP** | 4 | 1 | 3 | 1 | 3 | 0 | - |

**Table S3.** The number of enriched Gene Ontology (GO) terms and KEGG pathways in the VIGs of three coronaviruses and DGs of two neurodegenerative diseases. KEGG, KEGG pathway; BP, Biological Process; CC, Cellular Component; MF, Molecular Function.

| **Virus or Disease** | **KEGG** | **BP** | **CC** | **MF** |
| --- | --- | --- | --- | --- |
| SARS-CoV-2 | 61 | 1570 | 286 | 158 |
| SARS-CoV | 23 | 359 | 125 | 45 |
| MERS-CoV | 70 | 868 | 113 | 87 |
| AD | 176 | 3071 | 199 | 253 |
| PD | 155 | 2578 | 162 | 182 |

**Table S4**. The number of common genes between VIGs of three coronaviruses and DGs of two neurodegenerative diseases.

| **Virus** | **Number of genes overlapped between VIGs and DGs** | |
| --- | --- | --- |
|  | **AD** | **PD** |
| SARS-CoV-2 | 247 | 139 |
| SARS-CoV | 135 | 67 |
| MERS-CoV | 393 | 207 |
| Three viruses | 38 | 19 |

**Table S5**. The number of common KEGG pathways and GO terms enriched between the VIGs of three coronaviruses and DGs of two neurodegenerative diseases. K, KEGG pathway; B, Biological Process; M, Molecular Function; C, Cellular Component.

| **Virus** | **SARS-CoV-2** | | | | **SARS-CoV** | | | | **MERS-CoV** | | | | **Three viruses** | | | |
| --- | --- | --- | --- | --- | --- | --- | --- | --- | --- | --- | --- | --- | --- | --- | --- | --- |
|  | **K** | **B** | **M** | **C** | **K** | **B** | **M** | **C** | **K** | **B** | **M** | **C** | **K** | **B** | **M** | **C** |
| AD | 45 | 877 | 62 | 114 | 15 | 193 | 14 | 52 | 58 | 500 | 19 | 44 | 8 | 95 | 5 | 25 |
| PD | 37 | 815 | 45 | 88 | 12 | 198 | 13 | 43 | 56 | 468 | 19 | 42 | 7 | 97 | 5 | 22 |
| Both | 37 | 716 | 40 | 80 | 11 | 175 | 10 | 41 | 55 | 422 | 16 | 34 | 7 | 88 | 5 | 22 |

**Table S9.** The full name of viruses which interacted with seven genes.

| **Short name for virus** | **Full name of virus** | **Short name for virus** | **Full name of virus** |
| --- | --- | --- | --- |
| AAV | Adeno-associated virus | LCMV | Lymphocytic choriomeningitis virus |
| DENV | Dengue virus | LCV | La Crosse virus |
| Flu | Influenza virus | MeV | Measles virus |
| HAdV | Human adenovirus | MuHV | Murid herpesvirus |
| HCV | Hepatitis C virus | PV1 | Poliovirus type 1 |
| HDV | Hepatitis delta virus | ALV | Avian leukosis virus RSA |
| HHV | Human herpesvirus | RVFV | Rift valley fever virus |
| HIV | Human immunodeficiency virus | SV40 | Simian virus 40 |
| HPV | Human papillomavirus | ZEBOV | Zaire ebolavirus |
| MCPyV | Merkel cell polyomavirus | ZIKV | Zika virus |
| HRSV | Human respiratory syncytial virus |  |  |
